# When names are on the line: Negotiating authorship with your team

**DOI:** 10.1007/s40037-021-00675-8

**Published:** 2021-07-14

**Authors:** Glenn Regehr

**Affiliations:** Centre for Health Education Scholarship, Faculty of Medicine, University of British Columbia, Vancouver, BC Canada

In the Insider’s Perspective section, an insider in health professions education offers his/her thoughts, contemplations and advice on readers’ dilemmas or questions. You can send your questions or dilemmas to lieda.meester@bsl.nl. And who knows, your question may be the topic of the next instalment of the Insider’s Perspective.

*Dear Insider, when it comes to doing research with collaborative teams, I’ve experienced many different kinds of “collaboration.” Some collaborators engage deeply with the data and the analysis to really craft the story of the research. Some collaborators seem to be on the team primarily to provide access to participants or to a specific data set. Some collaborators are on the team as assistants and will really work in support—but they often don’t contribute new ideas during analysis meetings. Interestingly, there never seems to be a consistent understanding of what those different kinds of collaboration mean in terms of their status on the author line*.* Are there any rules to this? How do I manage these different ideas of what “collaboration” means and how that translates to recognition on the author line? I know the advice is to discuss it early in the collaboration, but what do I say?*

The official rules for authorship seem pretty clear in theory, but as always, the relationship of theory to practice can be pretty complex. Typically, the journals in health professions education reference the four criteria for determining authorship as set forth by the International Committee of Medical Journal Editors (ICMJE, http://www.icmje.org/recommendations/browse/roles-and-responsibilities/defining-the-role-of-authors-and-contributors.html):

- Substantial contributions to the conception or design of the work; or the acquisition, analysis, or interpretation of data for the work; AND
- Drafting the work or revising it critically for important intellectual content; AND
- Final approval of the version to be published; AND
- Agreement to be accountable for all aspects of the work in ensuring that questions related to the accuracy or integrity of any part of the work are appropriately investigated and resolved.

While these criteria might seem pretty straightforward, complexities can still arise because of discrepancies among members of the collaborative team regarding what constitutes “substantive contribution” or what qualifies as contributing “important intellectual content.” Personally, I see these criteria as more than just a checklist of activities; rather I think they are intended to represent the level of a person’s intellectual curiosity about, intellectual commitment to, and sense of intellectual “ownership” of the study and its discoveries. And these can be nebulous concepts and difficult to probe.

Thus, it might be helpful if you think of the authorship negotiation as an effort to understand each person’s motivation for participating in the research. Some, for example, may be invested in the research question itself, having a passion for advancing this area of work. Others may be committed to supporting research generally, and your study is just a place for them to do this. Still others might be particularly invested in supporting your career (as a mentor) or might be just offering some support to you as a favor to a colleague. And others might be taking a more transactional stance, such as a research assistant who is seeing participation in the work strictly as a source of gainful employment. This transactional motivation is particularly complicated by the fact that authorship in the academic world holds powerful symbolic capital, so some collaborators may be offering their support of the project as a way of “earning” authorship. These various motivations (which are not mutually exclusive) might affect the extent to which individuals on your “team” feel themselves to be meaningfully invested and engaged in the project and, in turn, “part owners” of the study, the ideas developed through the work, and the resulting paper.

My goal in this response will not be to make any judgements with regard to these motivations or the associated authorship claims. Nor will I offer any exact wording for starting such conversations, as these interactions always require nuanced adaptations to one’s culture, the local context, and the relationship you have established with the various team members. Instead, I will try to elaborate the sorts of potential considerations that your team members might be entertaining during your negotiations so that, in those discussions, you can look for what might be going on beyond simple interpretation of the criteria for authorship. These additional considerations may not always have perfect correspondence with the rules, but often they can be understood and managed if interpreted from the perspective that science is a social activity. Hopefully, this will allow you to approach the conversation in a nuanced way.

To explore these ideas, let’s start with an example from your question: the person who is recruited because of her ability to provide access to participants or a data set. Clearly the research could not have been conducted without this person’s active support, but such a role alone would likely not fulfill all the ICMJE criteria for authorship. Whether this becomes an authorship conflict might depend on how you position this person relative to the team and the research. For example, this person might be approached by the team once the study is well developed and asked whether she would be willing (based on a read of the study) to support the work by aiding in access to participants. By taking such an approach, you have positioned this person as a colleague who is helping you out rather than as a “member of the team.” So, from a transactional perspective, this sort of assistance might perhaps accrue a social debt, but this debt need not be paid with authorship on the resulting paper. By contrast, if the person is brought in early in the planning stages and therefore is positioned as being “on the team,” then a different social contract might be inferred, and the person might well assume that authorship is the mechanism by which she will be “compensated” for her support of the project. In this sense, the expectation of authorship for activities such as providing access to participants is something that you can manage by how you position the person relative to “the team” and the project.

Notably, if such a person does become an “assumed” author during early conversations, there may be ways to further engage the person such that they, in fact, more effectively satisfy the criteria of authorship. You might, for example, turn them into a key informant on your team who can help you interpret and understand your data from the perspective of someone on the ground. Or you might be able to leverage their arm’s length perspective at certain points in the data collection, data analysis, and write up, as a form of ongoing peer review to determine whether your argument is capturing their interest (as a proxy for the interest of your intended audience). This speaks to the importance of sharing early expectations (or aspirations) with regard to an individual’s positioning on the team, intellectual commitment to the work itself, and ultimately authorship. Determining these expectations as a part of your early conversations with team members allows you to decide whether a given person’s expectations constitute a situation you wish (or need) to accept and, if so, to manage the situation as well as possible throughout the study such that everyone is comfortable with the authorship decisions.

A similar sort of issue is relevant when considering another example from your question: the research assistant. Research assistants often join the team with the specific intent of receiving direct compensation for their activities, seeing the work as gainful employment. So, in that sense, the transactional aspect of the partnership is taken care of, and the decision regarding authorship might be more strictly held to the question of whether they are contributing intellectually (and are intellectually committed) to the work rather than just being “a pair of hands” and following instructions. In qualitative work this might be more complex as the research assistants are often involved in the coding process in ways that actually shape the codes. As a result, the line can be fuzzy and often it is other considerations that might further influence whether the person in question becomes an author. For example, if the research assistant aspires to an academic career, then a publication could have a strong influence on future success and there might be some consideration about helping to support the person’s efforts to enter academia. Again, knowing this early would allow the research team to change their expectations of the research assistant, requiring more “thinking work” and active participation in the writing of the paper, in order to legitimize the claim to authorship more fully, and avoid treating authorship as merely “doing them a favor.”

While these two examples have focused on how you might bring an individual who wishes to be an author into a more central intellectual role in the research enterprise, there are also times when a person who begins centrally on the team might drift to the periphery. Sometimes this arises because the tone or framing of a work as negotiated by the team starts to veer from a framing that a team member feels comfortable with, in which case the person might negotiate herself off the authorship and allow the team to pursue the work as they see fit. Other times, the person might simply become distracted with other projects or lose interest and choose to step away. Typically, these situations will be relatively straightforward, with everyone agreeing that the person would not be an author.

However, sometimes the situation is a bit more complicated. For example, a team member might be very committed to the study and wish to stay connected to the work, but life circumstances (such as illness or parental leave) require him to step back from full and active participation in the study. More awkward situations arise when a person who wishes to remain an active part of the team is inadvertently left out at critical stages. This last situation can happen, for example, when a subset of the team is in close proximity to each other and develop a pattern of work that leverages the proximity. A junior researcher may consistently approach the people around her such that formal research meetings don‘t happen and individuals more geographically distant find themselves on the periphery of the analysis and development of ideas until it is too late to contribute meaningfully. At this point, the peripheral team members might acknowledge that they do not qualify for authorship, but through no choice or fault of their own. How this is resolved will depend on the situation and the individuals involved. Sometimes the team may choose to revisit the analysis and writeup with the member re-engaged. Other times there may be an agreement that the excluded individual be left off authorship.

Even within a highly integrated and engaged team, however, there can be complexities in negotiating authorship. For example, considerations of authorship and author order on a given paper can be affected by the number of papers that are expected out of a program of research. Not all team members might be engaged in a particular analysis or the resulting paper. And even if so, there might be an explicit negotiation with regard to the rotation of authorship across a set of papers. In a PhD supervision, for example, if the supervisory team is all heavily active in the work, then there may be explicit decisions about rotating the last author (senior author) versus middle authorship so as to represent the equitable nature of the supervisory contributions. When done well, this rotation is not merely “in name” but rather the supervisory team might take turns in more actively mentoring the lead author in developing the ideas, and in shaping the analysis and the overall story of the paper (which, in my mind, defines the senior author role). This gives the student a broader experience of approaches to writing papers (much as rotating through clinics gives a resident a range of approaches that she can then incorporate into her own style of practice).

Additionally, there may be times when a member of the team offers extensive advice and intellectual input to the paper, thereby technically qualifying for authorship (perhaps even senior authorship), but the person might nonetheless choose to exclude her name from the authorship list. This might happen, for example, if a senior member of the team were to see her name on the paper as potentially accruing too much credit for the core idea (taking credit away from a burgeoning junior person). This is especially true if the paper is about an educational innovation that the lead author has been working on for an extended time. While the paper might be a collaboration, if the original innovation was not, then the supporting colleague might feel uncomfortable with the potential that she would receive implicit credit for the innovation. Sitting in this mentorship role, the person might be quite impressed with the work and be proud to have been a part of the enterprise but might nonetheless choose not to join the authorship list (or at least try to “hide” in a middle authorship position) in order to afford the more junior person appropriate prominence on the work.

Thus, as with all social interactions, the negotiation of authorship on papers has many complexities beyond simply asking each person to fill out the checklist of requirements for authors. An early conversation about this can, indeed, be helpful. But this opening conversation might be less about who wants to be an author (most are likely to say “yes” in the early discussions, both in order to keep the door open and because they are not thinking about how much work they might be committing to). Rather, the conversation might focus more on establishing perspectives, goals, and expectations as they are understood at the beginning of the enterprise. It might explore how engaged the potential team members are in the study itself and how involved they wish to be in various aspects of the work (what roles they wish to play). It is from this sharing of understandings and perspectives that authorship discussions can arise most effectively. And, as implied by these ideas, it is important to note that the authorship conversation is not a one-time event in the course of a study. Such conversations should be returned to at various points in the collaboration and may well continue right up to the completion of the paper itself. It is only through such iterative efforts that you can ensure that the final authorship decisions are as intellectually and emotionally satisfying as possible for everyone involved.

